# Inhibiting Angiogenesis by Anti-Cancer Saponins: From Phytochemistry to Cellular Signaling Pathways

**DOI:** 10.3390/metabo13030323

**Published:** 2023-02-22

**Authors:** Mohammad Bagher Majnooni, Sajad Fakhri, Syed Mustafa Ghanadian, Gholamreza Bahrami, Kamran Mansouri, Amin Iranpanah, Mohammad Hosein Farzaei, Mahdi Mojarrab

**Affiliations:** 1 Student Research Committee, Kermanshah University of Medical Sciences, Kermanshah 6714415153, Iran; 2 Pharmaceutical Sciences Research Center, Health Institute, Kermanshah University of Medical Sciences, Kermanshah 6734667149, Iran; 3 Department of Pharmacognosy, Isfahan Pharmaceutical Sciences Research Center, School of Pharmacy and Pharmaceutical Sciences, Isfahan University of Medical Sciences, Isfahan 8174673461, Iran; 4 Medical Biology Research Center, Health Technology Institute, Kermanshah University of Medical Sciences, Kermanshah 6714415185, Iran

**Keywords:** triterpenoid saponins, steroidal saponins, angiogenesis, VEGF, HIF-1α, inflammation, signaling pathways

## Abstract

Saponins are one of the broadest classes of high-molecular-weight natural compounds, consisting mainly of a non-polar moiety with 27 to 30 carbons and a polar moiety containing sugars attached to the sapogenin structure. Saponins are found in more than 100 plant families as well as found in marine organisms. Saponins have several therapeutic effects, including their administration in the treatment of various cancers. These compounds also reveal noteworthy anti-angiogenesis effects as one of the critical strategies for inhibiting cancer growth and metastasis. In this study, a comprehensive review is performed on electronic databases, including PubMed, Scopus, ScienceDirect, and ProQuest. Accordingly, the structural characteristics of triterpenoid/steroid saponins and their anti-cancer effects were highlighted, focusing on their anti-angiogenic effects and related mechanisms. Consequently, the anti-angiogenic effects of saponins, inhibiting the expression of genes related to vascular endothelial growth factor (VEGF) and hypoxia-inducible factor 1-α (HIF-1α) are two main anti-angiogenic mechanisms of triterpenoid and steroidal saponins. The inhibition of inflammatory signaling pathways that stimulate angiogenesis, such as pro-inflammatory cytokines, mitogen-activated protein kinase (MAPKs), and phosphoinositide 3-kinases/protein kinase B (PI3K/Akt), are other anti-angiogenic mechanisms of saponins. Furthermore, the anti-angiogenic and anti-cancer activity of saponins was closely related to the binding site of the sugar moiety, the type and number of their monosaccharide units, as well as the presence of some functional groups in their aglycone structure. Therefore, saponins are suitable candidates for cancer treatment by inhibiting angiogenesis, for which extensive pre-clinical and comprehensive clinical trial studies are recommended.

## 1. Introduction

Saponins are one of the most widespread secondary metabolites presents in the plant kingdom and marine organisms, generating stable foam in aqueous solutions, such as soap [1]. Humans have long used saponin-rich plants in their diets to maintain health and therapeutic benefits, including some Fabaceae plants [2], such as pea *(Pisum sativum*), beans (*Phaseolus* spp.), fava bean (*Vicia faba)*, soybean (*Glycine max*), mung bean (*Vigna radiate*), and licorice (*Glycyrrhiza glabra*). Additionally, *Allium* genus plants [3] are also rich in saponins, such as garlic (*Allium sativum*), onion (*Allium cepa*), and chives (*Allium schoenoprasum*). Moreover, herbs, including tea (*Camellia sinensis*), banana (*Musa* spp.), and ginseng (*Panax ginseng*), are found as saponin sources [4]. Saponins are composed of two parts, including aglycone (sapogenin) as non-polar moiety and glycoside (saccharide chain) as polar moiety. Triterpenoid saponins and steroidal saponins with, respectively, 30 and 27 carbon atoms, are the 2 main chemical structures of sapogenin core of saponins isolated from natural sources [5]. Furthermore, farnesyl diphosphate synthesized from the mevalonate biosynthetic pathway is the main precursor of the sapogenin [6,7]. Saponins have a high structural diversity due to the presence of functional groups on the sapogenin core, the number and type of sugars in the structure, and the way sugars bind to each other and to the aglycone part. Such structural diversity causes various biological and pharmacological effects [8]. Accordingly, several effects have been reported for saponins, such as anti-microbial, anti-leishmaniasis, anti-oxidant, anti-cholesterol, anti-lipidemia, analgesic, anti-inflammation, anti-diabetic, and anti-coagulant effects. [1,9]. Saponins also revealed the prominent anti-cancer and cytotoxic activities via regulating multiple signal pathways of cellular apoptosis, cell cycle arrest, proliferation, autophagy, and inflammation [10].

Angiogenesis, as the formation of new blood vessels, is one of the most important strategies to provide the energy needed for the survival and proliferation of cancer cells. Today, the inhibition of angiogenesis is used as a new and practical idea to control the growth and metastasis of tumors [11]. Since the discovery of the significant role of angiogenesis in the growth and progression of tumors by Folkman in 1971 [12], several drugs with anti-angiogenic effects have been introduced to the global pharmaceutical market, including bevacizumab, lenalidomide, axitinib, sorafenib, and everolimus. These drugs treat cancer by inhibiting the angiogenesis triggers, including the vascular endothelial growth factor (VEGF) and hypoxia-inducible factor 1-α (HIF-1α) release [13]. On the other hand, plants and their secondary metabolites have always been considered by researchers to treat cancer. According to investigations, plant secondary metabolites have also had a significant effect on inhibiting the growth and progression of tumors through blocking angiogenesis stimulation cellular signaling pathways [14]. Vinca alkaloids and paclitaxel, which have been isolated from *Catharanthus roseus* (Apocynaceae) and *Taxus brevifolia* (Taxaceae), respectively, are among anti-angiogenesis natural products found in pharmaceutical markets [15].

Sobolewska et al. reviewed the cytotoxic effects and anti-cancer mechanisms of saponins emphasizing the structure-activity relationship and their natural sources [16,17]. In this present review, the dramatic effects of saponins have been described on inhibiting the growth and metastasis of cancer via inhibiting the gene expression of angiogenesis mediators, including VEGF/VEGFR2, HIF-1α, fibroblast growth factor 2 (FGF2), and phosphoinositide 3-kinases/protein kinase B (PI3K/Akt). The anti-cancer effects of saponin was also shown to be applied through blocking inflammatory cellular signaling pathways, including pro-inflammatory cytokines, mitogen-activated protein kinase (MAPKs) pathways, and nuclear factor κ light chain enhancer of activated B cells (NF-κB). In this article, we aimed to review the chemical structure of saponins and their natural sources. Additionally, saponins with anti-angiogenic effects and their mechanisms are highlighted as promising candidates for cancer treatment.

## 2. Study Design

A comprehensive review was performed using electronic databases, including PubMed, Scopus, ScienceDirect, and ProQuest. The keywords ((“angiogenesis” OR “Neovascularization” OR “cancer” [title/abstract/keywords]) AND (“saponin” OR “steroidal saponin” OR “triterpenoid” [all fields]) were searched until December 2022. Only English language reports were included. After excluding duplicates, two independent researchers checked the primary retrieved results based on the title and abstract. Selected articles were then screened based on their full text. The whole in vitro, in vivo, and clinical trial studies in which the anti-angiogenesis effects and mechanisms of saponins were included. To complete the search strategy, the reference lists and citation hand search with the authors’ expertise in saponins as major therapeutic agents in angiogenesis were also used. Those reports included in the final article are summarized.

## 3. Angiogenesis: Cellular Signaling Pathways

The angiogenic process is a complex and extremely regulated action that enhances tumor progression and survival. Several signaling pathways and interconnected mediators are involved in the pathogenesis of angiogenesis that could be controlled through modulating multiple autophagy, oxidative stress, inflammatory, apoptotic pathways, and proangiogenic/antiangiogenic markers [18]. Pro-angiogenic mediators could be released through different kinds of cells, including endothelial cells (ECs), smooth muscle cells, fibroblasts, immune cells, platelets, and tumor cells [18,19]. The pro-angiogenic-signaling molecule VEGF and its cognate receptor, tyrosine kinase VEGF receptor 2 (VEGFR2), play a vital role in angiogenesis [20,21]. Epidermal growth factor (EGF), basic fibroblast growth factor (bFGF), platelet-derived growth factor (PDGF), transforming growth factor-β (TGF-β), angiopoietins, ephrins, apelin (APLN), granulocyte and granulocyte/macrophage colony-stimulating factors (G-CSF and GM-CSF), hepatocyte growth factor/scatter factor (HGF/SF), and inflammatory factors, including cytokines, such as interleukin-6 (IL-6), IL-1β, tumor necrosis factor-α (TNF-α), and chemokines are among other pro-angiogenic agents [19,21,22,23,24,25]. Inflammation is one of the main lethal factors in angiogenesis and cancer, which cause DNA damages through several macrophage migration inhibitory factor (MIF) and reactive oxygen species (ROS) and reactive nitrogen species (RNS) production [26]. These mediators contribute to the angiogenesis through the upregulation of transcription factors, such as MAPKs, NF-κB, signal transducer and activator of transcription 3 (STAT3), and mammalian target of rapamycin (mTOR). Recent studies demonstrated MAPK-signaling cascade and its main subfamilies, such as extracellular signal-regulated kinase (ERK), c-Jun NH2-terminal kinase (JNK), and p38 plays pivotal roles in inflammation/oxidative stress-associated angiogenesis. Besides phosphatases and tensin homolog (PTEN)/PI3K/Akt/mTOR, Janus kinase (JAK)/STAT and Ras/Raf/mitogen-activated protein kinase (MEK)/ERK/MAPK signaling pathways are also interconnected with the modulation of inflammation, oxidative stress, and cancer cells survival [27,28,29,30,31,32,33]. Oxidative stress is another trigger of angiogenesis that linked with aforementioned signaling pathways. Oxidative stress is caused by an imbalance between ROS/RNS production and antioxidants factors, including catalase (CAT), superoxide dismutase (SOD), glutathione peroxidase (GPx), and glutathione (GSH) [34]. Angiogenic inhibitors, including angioarrestin, angiostatin, endostatin, tumstatin, fibronectin fragment, IL-1, IL-12, interferons, canstatin, retinoic acid, arrestin, tissue inhibitors of metalloproteinases (TIMPs), multimerin 2, and angiogenic activators act together to maintain the balance of angiogenesis [13,19,23,35,36,37]. Angiogenesis is accomplished when stimulating factors, such as VEGF, angiopoietin-1 (ANG-1), and placental growth factor (PLGF), permeabilize the vessels. These factors also involved in the formation of new vessels. In addition, angiopoietin, angiogenin, TGF α/β, and matrix metalloproteinases (MMPs) are among other angiogenic stimulators [35,38,39]. Moreover, recent studies showed microRNAs (miRNAs) play a significant role in metabolism, apoptosis, protein secretion, cell proliferation, division, and differentiation that could modulate different stages of angiogenesis by targeting numerous genes within various signaling pathways. Thus, they could be new therapeutic targets in regulating angiogenesis [40]. In addition, hypoxia disrupts the balance between pro-angiogenic and antiangiogenic factors leading to the upregulation of HIF-1α, which suppresses antiangiogenic factors expression while promoting the pro-angiogenesis factors expression [19,41]. HIF-1α is the main stimulus for elevated VEGF, FGF, and PDGF production in cancer [23]. Hence, all the markers in the aforementioned signaling pathways could be considered effective therapeutic targets in angiogenesis.

## 4. Targeting Tumor Cells with Anti-Angiogenic Agents: Recent Advances

To stop angiogenesis, it is necessary to use anti-angiogenic factors or agents that decrease the production of pro-angiogenic markers, prevent them from binding to their receptors, or stop their functions [42]. Monoclonal antibodies (mAbs), small molecule inhibitors, VEGF inhibitors, tyrosine kinase inhibitors, gene therapy, and RNA interference (RNAi) therapy are auspicious anti-angiogenic interventions [19,43,44]. VEGF/VEGFR and interconnected downstream pathways have been the crucial targets of anti-angiogenic drugs. Small molecules inhibitors and mAbs modulating cell growth, differentiation, and angiogenesis through targeting VEGFRs, FGF receptors (FGFRs), EGFR receptors (EGFRs), PDGF receptors (PDGFR-α, PDGFR-β), Fms-like tyrosine kinase 3, and signaling proteins, including Raf, MAPK, mTOR, and PI3K [19,42,43,44]. Bevacizumab, aflibercept, and ramucirumab are monoclonal antibodies that exert anti-angiogenic effects by targeting the VEGF/VEGFR signaling pathway [21,43,44,45]. Receptor tyrosine kinase inhibitor small molecules (RTKIs) suppress signals inside blood vessels cells and are active against a numerous range of receptors involved in angiogenesis, including VEGFR, PDGFR, FGFR, rearranged during transfection (RET), and Tie receptors [43]. Sorafenib and sunitinib are among RTKIs that block angiogenesis and tumor growth via targeting the RAF/MEK/ERK signaling pathway, VEGFR-2, PDGFRB, and colony-stimulating factor-1 (CSF-1) [43,44]. The angiopoietins (Ang1–4)–Tie-axis is another pathway in angiogenesis. Ang1 and Ang2 expressions increased in many tumors. Ang1 binds to the Tie2 receptor and leads to a decline in vascular permeability and raised vessel stabilization, while Ang2 induces neovascularization and ECs migration and proliferation in response to pro-angiogenic markers [44,46,47]. Hence, a higher ratio of Ang1 to Ang2 predicts better outcomes. Trebananib showed promising antiangiogenics effects by targeting the Ang/Tie-axis in phase II trials [48]. Recent studies showed phytochemicals, such as alkaloids, saponins, tanshinone, coumarins, flavonoids and artemisinin, allow for the activity of modulating angiogenesis through regulating pro-angiogenic and anti-angiogenic factors [49,50]. In addition, vinca alkaloids (e.g., vinblastine, vincristine, vinorelbine, and vindesine), podophyllotoxin and its derivations (e.g., irinothecan, topothecan), taxanes (e.g., docetaxel, paclitaxel), camptothecins, and anthracyclines (e.g., daunorubicin, idarubicin, doxorubicin, and epirubicin) are effective herbal medicines on angiogenesis/cancer [19,24,49,50,51]. In recent decades, natural products have attracted great interest in combating tumor angiogenesis due to their fewer side effects and multi-functional mechanisms of action. Therefore, natural products and phytochemicals could be promising sources for the prevention or/and treatment of cancer.

## 5. Saponins

### 5.1. Chemistry, Biosynthesis, and Natural Sources

Saponins are a large group of secondary metabolites that are abundant in natural sources, including plants and marine organisms. These compounds have a high molecular weight, containing 27–30 carbons (C). In their structure, C3 is bound to the hydroxyl group in all aglycone saponins [52]. Saponins possess a polar/non-polar structure. In their polar sections, saponins have sugar chains (glycosylated forms), while the sapogenin structure makes the nonpolar part. Such chemical properties cause soap-like foams behaviors in aqueous solutions for saponins [53].

**Figure 1:**
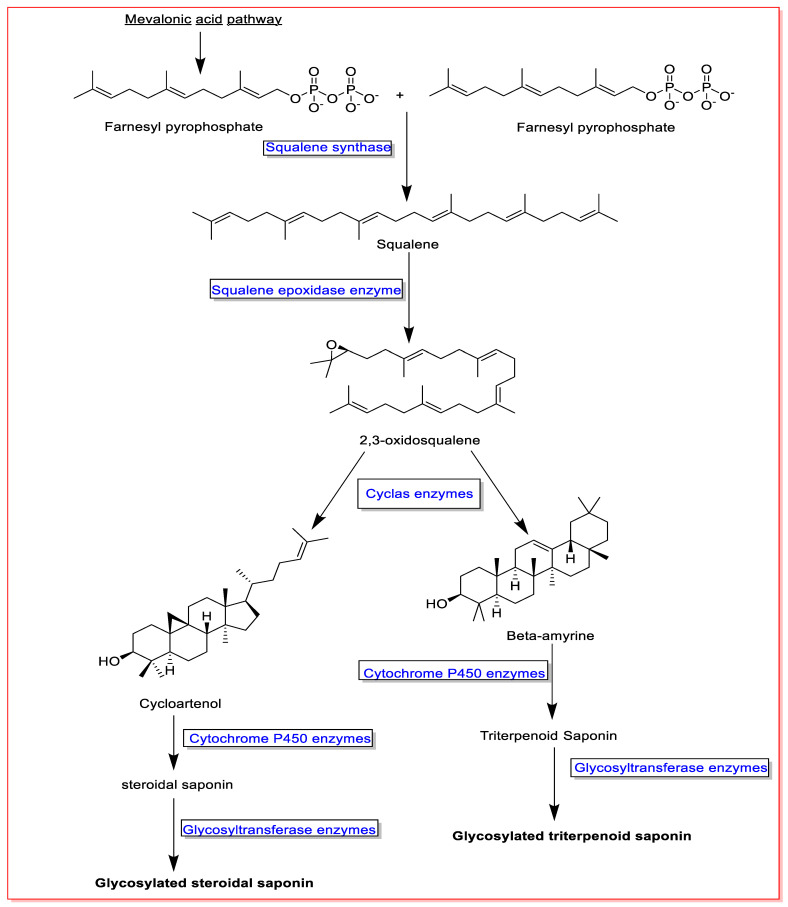
An overview on saponin biosynthesis pathway.

Squalene and 2,3-oxidosqualene are critical precursors in the biosynthesis of saponins. Accordingly, two units of farnesyl pyrophosphate formed in the mevalonate biosynthetic pathway and converted to squalene in the presence of the enzyme squalene synthase. The produced squalene is then converted to 2,3-oxidosqualene by the squalene epoxidase enzyme. Consequently, squalene and 2,3-oxidosqualene are converted to various aglycone saponins by different enzymes, cyclase and cytochrome P450. Finally, aglycone saponins are glycosylated by glycosyltransferase enzymes [6,7,54] (Figure 1).

**Figure 2:**
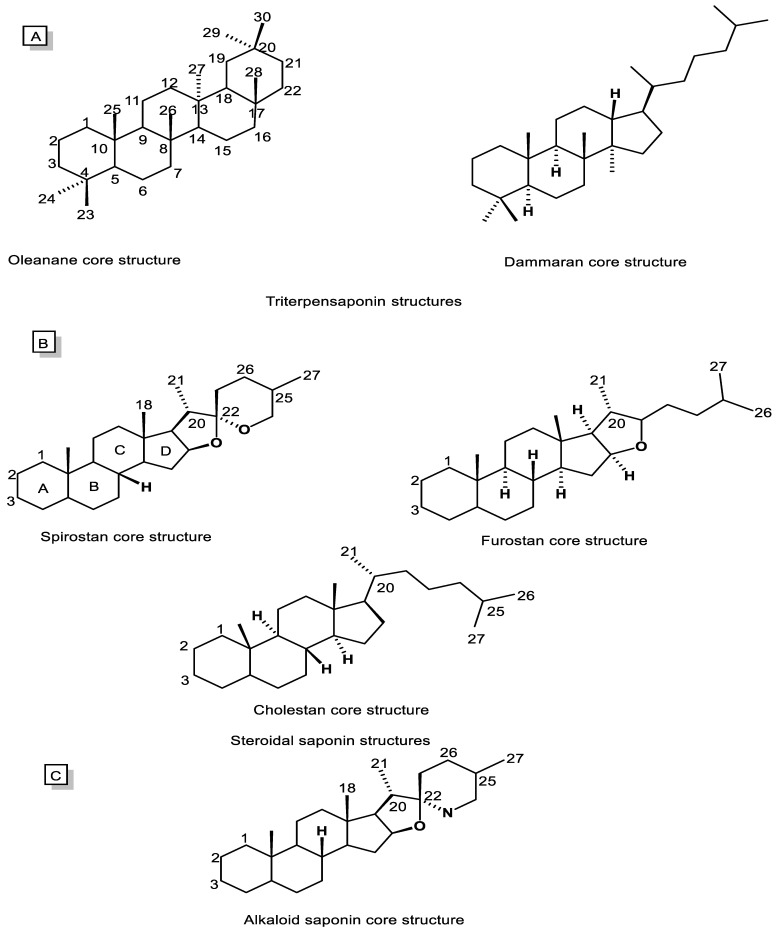
Chemical structures of saponin cores. (**A**) Triterpenoid saponins, (**B**) Steroidal saponins, and (**C**) Alkaloidal saponins.

Saponins are typically divided into two main categories, including triterpenoid saponins (C30, Figure 2A) and steroidal saponins (C27, Figure 2B). However, alkaloidal saponins (Figure 2C), which contain nitrogen atoms, are also classified in this category [5,55]. Consequently, steroidal saponins are divided into three categories, including spirostanes, furostanes, and cholestanes or open-chain steroidal saponins (Figure 2B). The spirostanes have a common spiro-carbon at the junction of two heterocyclic rings of furan and pyran in their structure and A and B rings are mainly *trans* isomer. The A and B rings of furostanes are found in both the *cis* and *trans* isomeric position and mainly consist of four hydrocarbon rings attached to a furan ring. Consistently, cholestane steroids do not have O-heterocyclic rings, and there is an opened ring in this group of saponins [52,56].

Oleananes and dammaranes (Figure 2A), with, respectively, five and four hydrocarbon rings, are two main groups of triterpenoid saponins isolated from natural sources, especially the plant kingdom. The terpenoid saponins rings are in an all-*trans* isomeric position. Additionally, their aglycone mostly contains functional groups including COOH, OH, and CH_2_OH at C28, C3, and C24, respectively [5,53,57].

Natural sources-isolated saponins are in glycosylated form and contain various saccharide chains. These saccharide chains are mainly oligosaccharides and composed of Dextro (D) or Leavo (L) isomers of sugars, such as glucose, galactose, rhamnose, arabinose, xylose, and glucuronic acid. However, sugars, such as quinovose, apiose, and fucose, are also found at a lesser frequency [53,58,59,60,61]. According to the number of saccharide chains attached to the saponin structure, these compounds are divided into two main categories: monodsmosidic and bidesmosidic, although in limited cases, tridesmoside saponins are also seen [62]. Although steroid saponins are generally monodsmosidic and are usually attached to the sugar chain at the C3 position, terpenoid saponins are bidesmosidic. Furthermore, tridesmoside, and the sugar chains, in addition to C3, are attached to the functional groups in C28 and C24 carbon. However, in some cases, the saccharide chain may bind to other carbons in the saponins structure [63,64,65].

Saponins are widely found in more than 100 families of dicotyledonous and monocotyledonous plants. These compounds are presented in different parts of the plants, including leaves, stems, roots, flowers, bark, and fruits, accumulate and thereby play protective roles against herbivores, microorganisms, and environmental stresses such as drought stress and temperature changes [66,67]. Steroid saponins are mostly found in monocotyledonous plants, such as Dioscoreaceae, Asparagaceae, Liliaceae, and Amaryllidaceae. Dicotyledonous families, such as Fabaceae, Araliaceae, Quillajaceae, Polygalaceae, Caryophyllaceae, Primulaceae, and Theaceae, mostly contain terpenoid saponins. Alkaloidal saponins are also isolated from the Solanaceae family [56,68,69]. Several steroidal saponins, such as dioscin, gracillin, deltonin, and parrisaponin, are isolated from *Dioscorea* genus [68]. Patricia Y et al. isolated shatavarin V as a new spirostanol saponin from the *Asparagus racemosus* (Asparagaceae) root [70]. Sobolewska et al. have introduced more than 350 steroidal saponins from *Allium* species (Liliaceae) in their review article [52]. In another study, 10 new terpenoid saponins were separated from the root of *Glycyrrhiza glabra* (Fabaceae), such as 11-deoxorhaoglycyrrhizin, 30-hydroxyglycyrrhizin, and 20α-rhaoglycyrrhizin [71]. Additionally, more than 100 saponins were isolated from *Quillaja saponaria* bark (Quillajaceae), with quillaic acid and its derivatives among the most well-known [72]. *Panax ginseng* (Araliaceae) is one of the rich sources of dammarane-type saponins, including ginsenoside derivatives [73]. Due to the presence of two or three hydroxyl groups in the chemical structure of ginsenosides, they are categorized into two main groups, including protopanaxadiol (Rb1, Rb2, Rg3, Rc, Rd, and Rh2) and protopanaxatriols (ginsenoside Rg1, Rg2, Rg3, Re, and Rh) [74,75]. Furthermore, nowadays, sea organisms, such as sea cucumbers, starfish, and sponges, have attracted the attention of researchers as rich sources of saponins [76,77,78]. Bahrami et al. isolated and identified 89 types of saponins, including terpenoid saponins, Holothurinoside and Holothurin derivatives, from a species of sea cucumber (*Holothuria lessoni*) [79].

In summary, due to their unique chemical structure and structural diversity, saponins have shown a bright future among natural compounds.

### 5.2. Pharmacological and Biological Activities

The structural diversity of saponins have led to their various biological and pharmacological effects. In addition to their biological effects, such as cytotoxicity [17], antibacterial [80,81], anti-viral [82], anti-fungal [83,84], anti-leishmania [85,86], anti-inflammation [87,88], and anti-oxidant [89,90] mechanisms, saponins have shown prominent pharmacological effects, such as cardioprotective [91], neuroprotective [92], anti-cancer [93,94], hepatoprotective [95], wound healing [96,97], analgesic [98,99], anti-rheumatoid [100,101] anti-convulsant [102] and immunomodulatory [103,104] activities. More recently, saponins have also been used as adjuvants in vaccines [105,106]. QS-21, isolated from *Quillaja saponaria* bark (Quillajaceae), is one of the saponins that has passed clinical trials in different phases for use as an adjuvant in vaccination [107]. Accordingly, studies showed the beneficial therapeutic effects of saponins on various diseases, including metabolic diseases, such as obesity [108,109], diabetes [110,111], hypercholesterolemia [112], hypertension [113,114], and osteoporosis [115].

Dutta et al. showed that rasmuside A, as a steroidal saponin isolated from *Asparagus racemosus* fruits, had an anti-leishmaniasis effect on the *Leishmania donovani* strain at 1.31 ug/mL [116]. The saponin-rich fraction of green tea seeds (*Camellia sinensis*, Theaceae) showed significant anti-bacterial effects against *Escherichia coli*, *Staphylococcus aureus* and *Salmonella* spp. via the penetration and destruction of the bacterial cell membrane/wall [117]. Another study showed anti-oxidant and anti-inflammation activities for *Camellia sinensis* root saponins [118]. Aginoside, as a spirostane saponin isolated from *Allium nigrum* (Liliaceae) bulbs, showed strong anti-fungal activities at 400 ppm against wild fungi, such as *Fusarium* spp., that invade plants [119]. Moreover, Li et al. showed that the saponin-rich extract of *Panax ginseng* steamed root ameliorated the ischemic injuries at 200 mg/kg and 400 mg/kg, orally via modifying the hemodynamic parameters, such as the left ventricular systolic pressure and heart rate, as well as a decreasing of the intracellular calcium ion overload [120]. Additionally, a clinical study has been designed on the healing effects of *Panax notoginseng* saponins on patients with hypertensive intracerebral hemorrhage (NCT02999048). Onjisaponin derivatives, as the triterpenoid saponins isolated from *Polygala tenuifolia* root (Polygalaceae), showed prominent neuroprotective effects and increased the survival of PC21 cell lines against glutamate-induced neurotoxicity [121]. Additionally, Meng et al. showed in vitro (0.5, 1, and 2.5 μM) and in vivo (2.5 mg/kg, 5 mg/kg, and 10 mg/kg, intragastrically, rats) potentials of Paris saponin VII as a steroidal saponin separated and purified from the rhizome of *Trillium tschonoskii* Maxim (Melanthiaceae). In their study, Paris saponin VII ameliorated rheumatoid arthritis by inhibiting the expression of inflammatory signaling pathways, such as mitogen-activated protein kinase, and reducing inflammatory cytokines [100]. On the other hand, the saponin-rich extract of sea cucumber (*Pearsonothuria graeff*) revealed marked anti-obesity and anti-hyperlipidemia effects in the rat fed with 0.08% saponin-rich extract and a high-fat diet for eight weeks [122]. Macrophyllosaponin B and astragaloside VII, as two terpenoid saponins found in *Astragalus* spp., showed regulatory effects on the immune system at 60 μg/albino mice intraperitoneal (i.p.) via blocking the activities of interlukin-4 and stimulating the expression of interlukin-2 and interferon-γ [123]. The saponin-rich fraction of *Clerodendrum infortunatum* leaves (Lamiaceae) showed anti-convulsant effects at 45 mg/kg, i.p., and relieved the central and peripheral pain at 35 and 40 mg/kg, i.p., respectively, in mice [124]. In addition, several clinical trials have been conducted on the beneficial therapeutic effects of saponins and plants rich in saponins, such as onion (*Allium cepa*, Araliaceae) and fenugreek (*Trigonella foenum-graecum*, Fabaceae) [110,125,126,127,128]. So far, several clinical trials have been reported on the therapeutic effects of ginsenoside saponins on various diseases, including cardiovascular disorders, diabetes, blood lipids, high blood pressure, and obesity [129,130].

Furthermore, saponins have shown significant cytotoxic and anti-cancer activity through various mechanisms in in vivo and in vitro studies, which we will discuss in more detail in the next section. Overall, saponins have high therapeutic potential and could be introduced as promising candidates for the treatment of complex diseases, such as metabolic disorders and cancer.

### 5.3. An Overview on the Anti-Cancer Mechanisms of Saponins

According to the World Health Organization, cancer is rapidly spreading worldwide and has been the main cause of death in more than 60% of countries (112 out of 183 countries studied) in the world [131]. Therefore, researchers in the field of cancer are trying to find effective treatments for it. Due to their low side effects, multi-therapeutic targeting, and in most cases, their low-cost phytochemicals have been promising candidates for cancer treatment. Among the phytochemicals, saponins have been prominent anti-cancer candidates in vitro and in vitro [10,94].

#### 5.3.1. Anti-Cancer Activity of Steroidal Saponins

Steroidal saponins showed anti-cancer activities on several tumor cells via different mechanisms, including downregulating MAPKs, PI3K/Akt/mTOR, MEK/ERK1/2, and Bcl-2 [10,17].

PI3K/Akt/mTOR is a critical signaling pathway in controlling cell division, survival, differentiation, and metabolism. The dysregulation of this pathway in several cancer types occurs and causes cancer cell growth, angiogenesis, metastasis, and cell migration. On the other hand, enhancing the gene expression of PI3K/Akt/mTOR mediators increases cancer cells’ resistance to chemotherapy. Therefore, the inhibition of such pathways could be one of the pivotal strategies for cancer treatment [132,133]. Timosaponin AIII, as a glucosylated spirostane saponin of *Anemarrhena asphodeloides* (Asparagaceae) rhizomes, combated taxol-resistant lung cancer cells in vitro (2–8 μM) and in vivo (2.5 and 5 mg/Kg) via reducing PI3K/Akt/mTOR gene expressions [134]. As a steroidal saponin isolated from *Aspidistra letreae* (Asparagaceae), aspiletrein A showed cytotoxic effects on human lung cancer, including H460, H23, and A549 at 6.25 μM and 12.5 μM through inhibiting the expression of Akt [135]. Diosgenin as a spirostanol saponin found in *Trigonella foenum graecum* (Fabaceae) showed prominent anti-cancer activities with different mechanisms, including downregulating PI3K/Akt/mTOR gene expression [136]. DG-8d, as a synthetic derivative of diosgenin, obstructed PI3K/Akt signaling pathway at 20 μM, thereby inhibiting the growth of non-small cell lung cancer cell lines [137]. In another study, A-24 (2–8 μM) as a spirostanol saponin of *Allium chinense* (Liliaceae) bulbs revealed anti-proliferation effects through blocking the PI3K/Akt/mTOR cellular signaling pathway on SGC-7901 and AGS, as human gastric adenocarcinoma cell lines [138].

The Bcl-2 family plays a critical role in apoptosis. Bax and Bcl-2 are two vital members of the Bcl-2 family. In this line, Bax induces apoptosis, and Bcl-2 prevents cell death by blocking the Bax activities. Therefore, the Bax/Bcl-2 ratio is a critical factor for the determination of anti-apoptosis activities of anti-cancer agents in vitro and in vivo studies [139,140]. Zingiberensis, as a glycosylated steroidal saponin isolated from *Dioscorea zingiberensis* rhizomes *(Dioscoreaceae)*, showed cytotoxic activities on ovarian (SK-OV-3) and lung cancer (C26) cell lines at 1.51 and 0.81 μM, respectively. They found that reducing the Bcl-2 gene expression was the main anti-cancer mechanism of zingiberensis [141]. Raju et al. showed that feeding with 1% (*w*/*w*) and 0.05% (*w*/*w*) of diosgenin significantly inhibited the growth of colon cancer at 42% and 24%, respectively, in F344 rats. The anti-cancer effect was applied by reducing Bcl-2 gene expression and enhancing caspase-3 gene expression as two signaling pathways of apoptosis induction [142].

Glycolysis and oxidative phosphorylation are pivotal sources of energy for cancer cells and thereby play a critical role in energy production for cancer cells and their survival [143]. Thus, employing the inhibitors of such metabolic pathways could be introduced as potential anti-cancer compounds [144]. Gracillin (5 μM), as a spirostanol saponin isolated from *Dioscorea collettiivar* rhizomes (Dioscoreaceae), inhibited the growth of breast cancer tumor via blocking the oxidative phosphorylation and glycolysis [145].

Overgeneration of ROS induces cell cycle arrest, apoptosis, and autophagy in cancer cells. Therefore, stimulation of ROS production is one of the important strategies in chemotherapy and the discovery of anti-cancer agents [146,147]. Spirostane saponins, SAP-1016 and SAP-884, and furostane saponins, KE-1046 and KE-1064, showed cytotoxic activities against colon and breast cancer cell lines. SAP-1016 at 0.05 μM revealed more potential anti-cancer activities via enhancing ROS production [148]. Macrostemonoside (100 μM), found in *Allium macrostemon* (Liliaceae) bulbs, is another spirostane saponin with anti-colorectal cancer activities by increasing ROS production [149].

The steroidal saponins (asterosaponins) of a starfish found in Pacific and Indian oceans, *Acanthaster planci* (Acanthasteridae), revealed cytotoxic effects on HT-29 and MDA-MB-231 at 10 μM [150]. Paris saponin VII [151], a steroidal saponin of *Trillium tschonoskii* rhizomes and roots (Melanthiaceae), total soy saponins [152] of *Glycine max* fruits (Fabaceae), protoneogracillin [153], a furostanol saponin of *Dioscorea collettiivar* rhizomes (Dioscoreaceae), and hecogenin and tigogenin [154], spirostane saponins of *Agave tequilana* (Asparagaceae) are among other anti-cancer steroidal saponins. According to the previous reports, increasing the length of the sugar moiety and its binding site in steroidal saponins can be related to enhancing their anti-cancer effects [155].

#### 5.3.2. Anti-Cancer Activity of Triterpenoid Saponins

Triterpene saponins showed prominent anti-cancer activities. Inhibition of PI3K/Akt/mTOR, MAPKs, Wnt/β-catenin, and inducing ROS production are the critical anti-cancer mechanisms of triterpenoid saponin [10,155].

Afrocyclamin A, a triterpene saponin found in *Androsace umbellata* (Primulaceae), induced cell cycle arrest and inhibited cell migration in prostate cancer via reducing mTOR/PI3K/Akt at 4 μM and 8 μM. Moreover, afrocyclamin A (5 mg/kg, orally, 4 weeks) inhibited prostate tumor growth in mice [156]. Ginsenoside Rg3, as another triterpene saponin found in *Panax ginseng*, showed anti-lung cancer effects at 50, and 100 μg/mL via suppressing PI3K/Akt [157]. Additionally, a meta-analysis study of clinical trials conducted on the combined treatment of ginsenoside Rg3 with first-line chemotherapy drugs confirmed its beneficial effects in non-small cell lung cancer [158]. Furthermore, Platycodin-D, as a triterpene saponin isolated from *Platycodon grandiflorum* (Campanulaceae), obstructed PI3K/Akt/mTOR signaling pathways, thereby showing robust anti-lung cancer effects [159].

MAPK signaling pathways are complex and made of kinases, which associated regulation plays a major role in cell death, cell migration and invasion, angiogenesis, and progressing cancer cells. In fact, the downregulation of MAPK pathways and its downstream is one of the main mechanisms of anti-cancer agents [160,161]. Peng et al. showed that the total saponins of *Conyza blini* (Asteraceae) significantly inhibited the growth of cervical carcinoma tumor via downregulating the MAPKs/transforming growth factor beta (TGF-β)/nuclear factor erythroid 2-related factor 2 (Nrf2) as a vital cellular signaling pathway of inflammation and cell proliferation at 15 μg/mL and 20 μg/mL. The liquid chromatography-mass spectroscopy analysis of *Conyza blini* saponins confirmed the presence of triterpenoid saponins, such as 2β, 16α, 23-trihydroxyoleanolic acid, and 2β, 23-dihydroxyoleanolic acid [162]. Triterpenoid saponins isolated from *Glycyrrhiza glabra* root (Fabaceae) showed anti-cancer effects on breast, cervical, liver, pancreatic, colorectal, lung, gastric, and other types of malignancies via downregulating the PI3K/Akt, MAPKs signaling pathways [163].

Inhibiting Wnt/β-catenin cellular signaling pathways is another anti-cancer mechanism of saponins. This signaling pathway plays a critical role in cell proliferation, migration, and apoptosis. 1C (25 mM) as a ginsenoside saponin found in *Panax ginseng* (Araliaceae) revealed anti-prostate cancer activities via inhibiting the Wnt/β-catenin signaling pathway [164]. Zafar et al. reported that tubeimoside-1, a triterpenoid saponin found in *Bolbostemma paniculatum* (Cucurbitaceae), has broad anti-cancer effects on several cancer cell lines, such as the pancreas, esophageal, gastric, neuroblastoma, and bladder via regulating the gene expression of different signaling pathways involved in cell cycle arrest, apoptosis, and inflammatory cytokine generation [165]. Furthermore, tubeimoside-1 showed anti-colon cancer effects at 10, 25, and 50 μg/mL through inhibiting Wnt/β-catenin [166].

Additionally, increasing reactive oxygen species (ROS) generation is another 1C anti-cancer mechanism [164]. Moreover, Lin et al. reported that glycyrrhizic acid as one the main triterpenoid saponins of *Glycyrrhiza glabra* root inhibited breast cancer via enhancing ROS production [167].

Cercodemasoide derivatives, as the triterpenoid saponins separated and purified from sea cucumber *Cercodemas anceps* (Cucumariidae), showed prominent cytotoxic effects. In their study, cercodemasoide A killed the human hepatocellular carcinoma (Hep-G2) at 0.07 μM, while ellipticine, as a positive control, killed this cell line at 1.71 μM [168]; polyacanthoside A (*Acacia polyacantha* leaves, Fabaceae) [169], hederagenin (*Clematis ganpiniana* whole plant, Ranunculaceae) [170], and saikosaponins (*Bupleurum chinense* roots, Apiaceae) [171] are other anti-cancer triterpenoid saponins.

**Figure 3:**
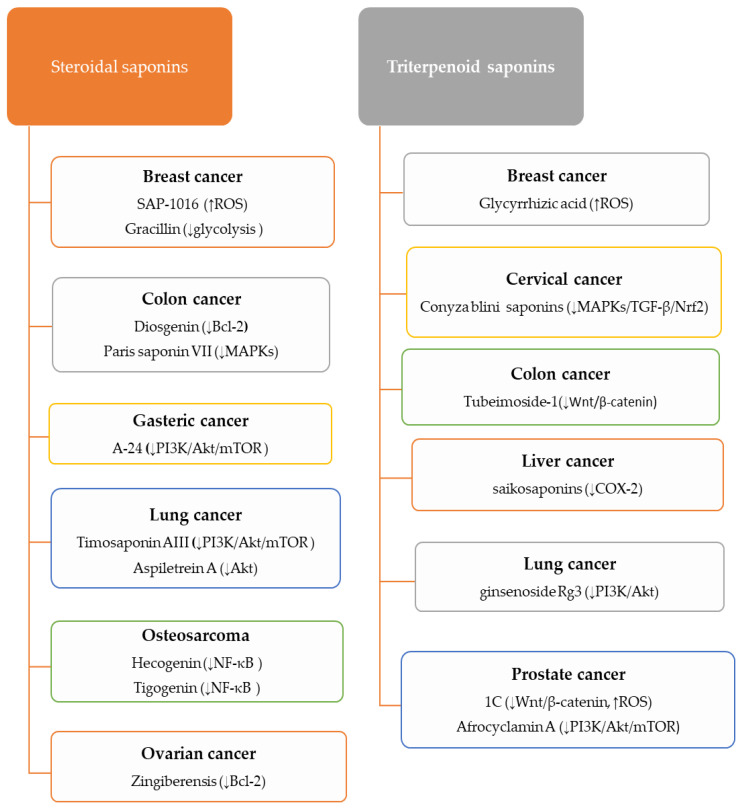
Anti-cancer activities of some steroidal and triterpenoid saponins.

In summary, saponins exhibit significant anti-cancer activities against various malignancies by regulating apoptotic and inflammation signaling pathways and inducing cell cycle arrest (Figure 3). Therefore, they have always been considered promising candidates for cancer treatment. On the other hand, the inhibition of angiogenesis in tumors as another main mechanism of saponins anti-cancers has attracted the attention of many researchers.

### 5.4. Anti-Angiogenic Potentials

The inhibition of angiogenesis and direct killing of cancer cells are two main strategies in the treatment of cancer [172]. Ever since Folkman proposed the theory of angiogenesis inhibition for the treatment of cancer in 1971, many efforts have been made to find molecules with anti-angiogenic effects [12]. These investigations led to the introduction of some drugs, such as sorafenib, bevacizumab, sunitinib, everolimus, and axitinib, with anti-angiogenic effects in the treatment of various cancers [13]. Natural sources, especially phytochemicals, have been among the main candidates for the discovery of molecules with anti-angiogenic effects [173]. Alkaloid-based drugs (e.g., vincristine, vinblastine, and camptothecin) [174], terpenoid-based drugs (e.g., paclitaxel) [175], flavonoid-based drugs (e.g., naringenin, kaempferol, and hesperidin) [176], and coumarin-based drugs (e.g., galbanic acid, esculetin, and daphnetin) [19] are among the major phytochemicals reported to inhibit angiogenesis. Additionally, Saponins, as one of the largest groups of natural compounds, have shown notable anti-angiogenic effects in the treatment of various cancers, in vitro and in vivo.

#### 5.4.1. Anti-Angiogenic Potentials of Steroidal Saponins

**Figure 4:**
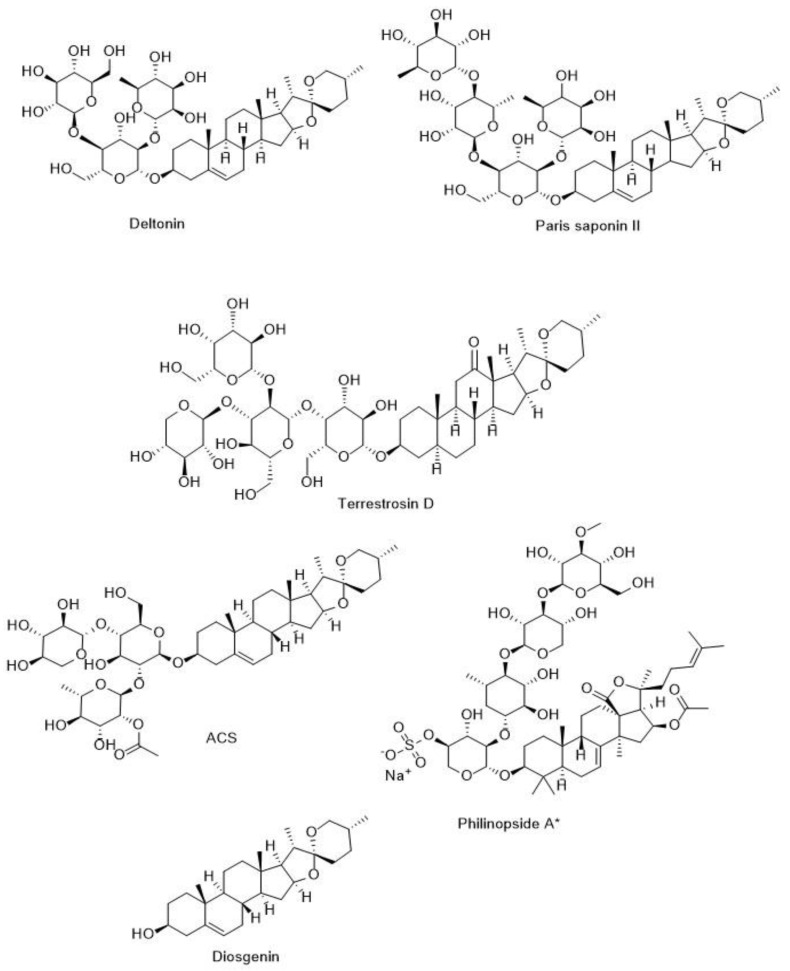
Chemical structure of some anti-angiogenic steroidal saponin. * Philinopside A: is a sulfated saponin isolated from marine organism.

The inhibition of angiogenesis is one of the critical anti-cancer mechanisms of steroidal saponins. Diosgenin (5, 15, and 25 µM), as a steroidal saponin found in *Dioscorea* spp. root (Dioscoreaceae) and *Trigonella foenum-graecum* (Fabaceae) seeds, reduced the level of two main mediators of angiogenesis (VEGF and FGF2) in glioblastoma cell lines [177]. In addition, dioscin and deltonin (Figure 4), as two glycosylated form of diosgenin found in *Dioscorea* spp. (Dioscoreaceae), inhibited angiogenesis induced by VEGF (50 ng/mL) in HUVEC via down-regulating the VEGFR2 downstream pathways, including MAPK/Akt pathways at 1 and 4 μM [178,179]. The sugar moiety of dioscin consists of two rhamnose units and one glucose unit, while the sugar chain of deltonin consists of two glucose units and one rhamnose unit. Investigations showed that the presence of more rhamnose units in the sugar moiety of steroidal saponins enhanced their anti-cancer activities [17]. Additionally, a steroidal saponin of *Tribulus terrestris* (Zygophyllaceae), terrestrosin D (Figure 4), showed significant anti-angiogenesis activities at 50 mg/kg in male Balb/c mice with prostate cancer. In their study, contrary to expectations, terrestrosin D increased the level of VEGF in HUVEC, so direct endothelial cell destruction by this saponin was suggested as a possible mechanism of its anti-angiogenic effect [180]. Steroidal saponins from the rhizome of *Polyphylla paris* (Melanthiaceae), including Paris saponin I, II, Ⅵ, and Ⅶ, blocked induced VEGF angiogenesis in HUVEC vascular endothelial cells at 2 and 4 μM. Furthermore, polyphyllin D, as another *Polyphylla paris* steroidal saponin, prevented angiogenesis in zebrafish embryos model at 0.313 μM and 0.156 μM. These saponins inhibited the critical downstream pathways of angiogenesis, including PI3K/Akt/mTOR/ribosomal protein S6 kinase beta-1 (S6K1), SRC/endothelial nitric oxide synthase (eNOS), phospholipase Cγ (PLCγ)/ERK, Janus kinase 2 (JAK2)/signal transducer and activator of transcription 3 (STAT3), and VEGFR2 [181,182]. Moreover, Paris saponin II (Figure 4) revealed anti-cancer activities against ovarian cancer via blocking NF-κB as one of the signaling pathways of inflammation and VEGF stimulant generation, thereby inhibiting angiogenesis in in vitro (2.5 μM) and in vivo (25 mg/kg, i.p., female Balb/c mice) [183,184]. The gene expression of MMPs as one of the critical triggers of angiogenesis was suppressed by ACS (Figure 4), a steroidal saponin isolated from *Ophiopogon japonicas* roots (Asparagaceae) at 1.25, 2.5 and 5 μM in HUVECs and C57/BL mice [185]. ERK1/2, HIF-1α and Akt are stimulated under hypoxia conditions and caused the release of VEGF, thereby initiating angiogenesis. These signaling pathways were inhibited in HUVEC by DT-13 as another steroidal saponin of *Ophiopogon japonicas* roots (Asparagaceae) at 0.01, 0.1, and 1 μM [186]. In addition, the steroidal saponin of *Convallaria majalis* rhizome (Asparagaceae), convallamaroside, reduced neovascularization in mice with kidney cancer at 5–100 µg/day [187]. In summary, the steroidal saponin revealed prominent anti-angiogenesis activities by inhibiting the angiogenesis cellular signaling pathways, including PI3K/Akt/mTOR, MAPKs, and JAK/STAT, that were stimulated with VEGF. The studies confirmed sugar moiety attached to C-3 increased the steroidal saponins anti-cancer and anti-angiogenesis activities.

#### 5.4.2. Anti-Angiogenic Potentials of Triterpenoid Saponins

**Figure 5:**
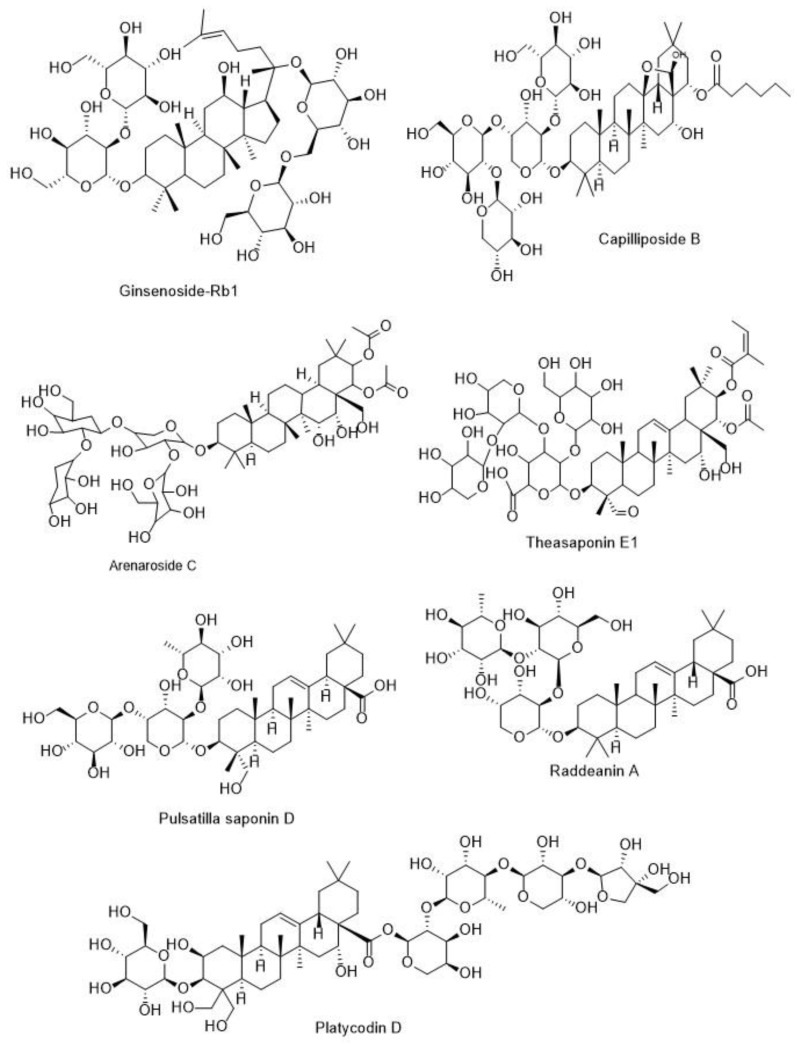
Chemical structure of some anti-angiogenic triterpenoid saponins.

Triterpenoid saponins have shown remarkable anti-angiogenic activities by modulating different cellular signaling pathways, which are discussed below. AG36, as a triterpenoid saponin isolated from *Ardisia gigantifolia* (Primulaceae) rhizomes, inhibited breast tumor growth in female BALB/c mice at 0.75 and 1.5 mg/kg/day through anti-angiogenic effects. In their study, AG36 (20 5, 10, and 20 μM) inhibited VEGF and its receptor, as well as focal adhesion kinase (FAK)/PI3K/Akt cascade, as stimulators of VEGF production. According to a docking study, the presence of a four-sugar chain was necessary for strong interaction between AG36 and VEGFR2 [188]. Arenaroside derivatives (Figure 5), including Arenaroside C, D, E, and G, as the polyhydroxylated triterpenoid saponins isolated from *Polycarpaea arenaria* (Caryophyllaceae), showed anti-angiogenic effects at doses less than 5 μM. In their study, it was found that the presence of the acetyl group at C-22 of polyhydroxylated triterpenoid saponins led to more anti-angiogenic effects [189]. Furthermore, ginsenosides are the dammarane type of triterpenoid saponins that are found in the organs of the *Panax* spp. (Araliaceae), especially *Panax ginseng* roots. The ginsenosides revealed significant anti-angiogenesis activities in several reports [190,191]. In this line, ginsenoside-Rb1 (Figure 5) inhibited angiogenesis in HUVEC at 100–500 nM by enhancing the gene expression of pigment epithelium-derived factor (PEDF), as the most abundant endogenous anti-angiogenesis glycoproteins [192]. Additionally, Lu et al. showed that ginsenoside-Rb1 upregulated the PEDF gene expression via blocking the microRNA-33a (miR-33a) as one of the main miRNAs of angiogenesis activator. In their study, it was found that ginsenoside-Rb1 inhibited miR-33a by increasing the expression of peroxisome proliferator-activated receptor-γ (PPAR-γ) and PEDF. Since ginsenosides have steroid-like rings in their chemical structure, in their study, the role of steroid receptors was confirmed in the regulation of miR-33a and PEDF [193]. Ginsenoside-Rb2 prevented angiogenesis in C57BL/6 mice with melanoma at 100 µg/mouse, intravenously [194]. Of other ginsenosides, ginsenoside K is one of the active deglycosylated metabolites of ginsenoside Rb1, which is metabolized by the intestinal bacterial flora after the oral ingestion of *Panax ginseng* roots [195]. Ginsenoside K abrogated the activities of sphingosine kinase 1 (SPHK1), as an angiogenesis mediator enzyme, in HUVEC at 10 µg/mL. The higher lipophilicity of ginsenoside K is related to its aglycone structure, and this is probably the reason for its strong affinity for SPHK1 [196]. Ginsenoside Rg3 (20 mg/kg), alone and in combination with gemcitabine, inhibited angiogenesis in female C57BL/6 mice with lung cancer by reducing VEGF gene expression [197]. Moreover, senegin III (0.1, 1, and 10 μM), as a triterpenoid saponin of *Polygala senega* roots (Polygalaceae), inhibited VEGF-induced angiogenesis by increasing PEDF. This triterpenoid saponin obstructed the angiogenesis induced by bFGF (200 ng/mL, subcutaneously) at 1 and 2 mg/Kg (i.p.; 10 days) in mice. The results confirmed that the presence of the C-28 sugar chain was necessary for its anti-angiogenesis activities [198]. Oldhamianoside II (5, 10, and 20 mg/kg), a bidesmosidic oleanolic acid derivative saponin of *Gypsophila oldhamiana* roots (Caryophyllaceae), markedly revealed anti-cancer activities against sarcoma and ovarian cancers via blocking the multi-targets of angiogenesis pathways, including VEGF and VEGFR2, COX-2, bFGF, and pro-inflammatory cytokines, such as TNF-α and IL-6 [199,200]. A triterpenoid saponin isolated from *Camellia sinensis* seeds (Theaceae), theasaponin E1 (2 μM and 10 μg/mL), showed anti-angiogenesis activities by decreasing the gene expression of VEGF, Akt, and HIF-1α. Theasaponin E1 (Figure 5) contains a four-sugar chain attached to C-3, and studies indicated that this chain is necessary for anti-angiogenesis activities [201,202]. Chiisanoside, a triterpenoid saponin isolated from *Acanthopanax sessiliflorus* (Araliaceae) leaves, reduced the VEGF plasma level in (H22) tumor-bearing mice at 60, 120, and 240 mg/kg, and in this way, it suppresses angiogenesis and tumor growth [203]. Additionally, Pulsatilla saponin D (Figure 5), as a triterpenoid saponin of *Pulsatilla koreana* (Ranunculaceae) roots, inhibited gene expression of the promotion angiogenesis mediators, including VEGF and HIF-1α at 10 μM in human liver, pancreatic, gastric, and colon cancer cell lines. The structure–activity relationship studies of Pulsatilla saponins showed that the presence of the carboxyl group at the C-28 position was necessary for the anti-cancer activities of these saponins, although it increased their hemolytic effects [204,205,206,207,208]. Platycodin D (Figure 5) [209,210], julibroside J8 [211], maesasaponins (I-VII.1) [212], astragaloside IV [213], capilliposide B (Figure 5) [214], raddeanin A (Figure 5) [215,216], and gleditsiosides B [217] are other triterpenoid saponins with anti-angiogenesis activities described in Table 1. Triterpenoid saponins showed anti-angiogenesis activities via blocking the pro-angiogenesis mediators, including VEGF and HIF-1α, as well as suppressing the PI3K/Akt, MAPKs, and NF-κB as stimulation angiogenesis cellular signaling pathways. Furthermore, these saponins increased the PEDF as an endogenous anti-angiogenesis protein. Investigations indicated the presence of sugar moiety in C-3 and C-28, and the carboxyl and acetyl functional groups at C-28 and C-22, respectively, enhances the triterpenoid saponin anti-angiogenesis and anti-cancer activities.

#### 5.4.3. Anti-Angiogenic Potentials of Marine Organism Saponins

Isolated saponins from marine organisms showed anti-angiogenesis activities [218]. Philinopside E, as a sulfated triterpenoid saponin of sea cucumber *pentacta quadrangularis* (Cucumariidae), inhibited angiogenesis in vitro (1.25, 2.5, and 5 μM) and in vivo (2 mg/kg and 3 mg/kg, intravenously, female KM mice) studies. In their study, reduction in the gene expression and activities of angiogenesis mediators such as kinase insert domain receptor (KDR)/VEGFR2 had been reported as the main anti-angiogenesis pathway of Philinopside E [219,220]. Philinopside A (IC_50_, 50 μM), another sulfated triterpenoid saponin (Figure 4) of *pentacta quadrangularis* (Cucumariidae), blocked all angiogenesis-mediated receptor tyrosine kinases (RTKs), such as VEGFR2 and platelet-derived growth factor receptor-β (PDGFR-β) [221]. On the other hand, triterpenoid saponin isolated from sea cucumber *Cucumaria frondosa* (Cucumariidae), frondoside A, revealed anti-angiogenesis activities through inhibition bFGF activities at 100 and 500 nM, thereby reducing lung tumor growth in NMRI female mice [222]. Furthermore, the total saponin of sea cucumber, *Holothuria leucospilota*, suppressed the VEGF gene expression at 3, 6, or 12 µg/mL in breast cancer cell lines [223]. These studies showed that the marine organisms can be introduced as new sources for discovering anti-angiogenesis saponins.

#### 5.4.4. Anti-Angiogenic Potentials of Total Saponin Extraction

The total saponin extraction of plants showed prominent anti-angiogenesis activities. Ahmad et al. showed that the total saponin extract of *Rumex hastatus* (Polygonaceae) aerial parts suppressed angiogenesis at IC50 64.9 μg/mL in the chorioallantoic membrane (CAM) angiogenesis model [224]. The crude Astragalus saponins extracted from *Astragalus membranaceus* (Fabaceae) exhibited anti-cancer effects against colon and gastric cancer at 80 and 50 μg/mL, respectively, via inhibiting angiogenesis-inducing signaling pathways, including VEGF, bFGF, HIF-1α, and PI3K/mTOR/Akt [225,226]. Additionally, the crude saponins extract from *Actinidia valvata* (Actinidiaceae) roots inhibited the gene expression of VEGF and bFGF, thereby revealing anti-angiogenesis activities in hepatoma H22 mice at 1 g/kg/d [227]. In another study, Albiziasaponins of *Albizia lebbeck* (Fabaceae) showed anti-angiogenesis activities at 0.5, 0.1, and 1 µg/mL in the CAM model [228]. Moreover, the total saponins of sea cucumber, *Holothuria leucospilota*, suppressed the VEGF gene expression at 3, 6, or 12 µg/mL in breast cancer cell lines [223]. Regarding the excellent anti-angiogenic effects of extracts rich in saponins, it can be concluded that the presence of several saponins with different anti-angiogenic cellular mechanism targets has made the synergy of their anti-angiogenic effects.

In summary, saponins showed notable anti-angiogenesis activities via several mechanisms, including suppressed gene expression involved in the release and activation of VEGF, such as PI3K/mTOR/Akt, HIF-1α, bFGF, JAK/STAT, VEGFR2, and MAPK pathways. Additionally, inhibiting the gene expressions of pro-inflammatory cytokines including COX-2, TNF-α, and IL-6, enhancing the PEDF gene expression, and direct inhibitory effects on vein endothelial cell proliferation are other anti-angiogenesis mechanisms of saponins. Furthermore, studies showed the direct relationship between chemical structure and saponin anti-angiogenesis activities. Finally, due to the prominent anti-angiogenic activities of triterpenoid and spirostanol saponins (Figure 4 and Figure 5), these compounds can be introduced as promising anti-cancer candidates.

## 6. Conclusions

**Figure 6:**
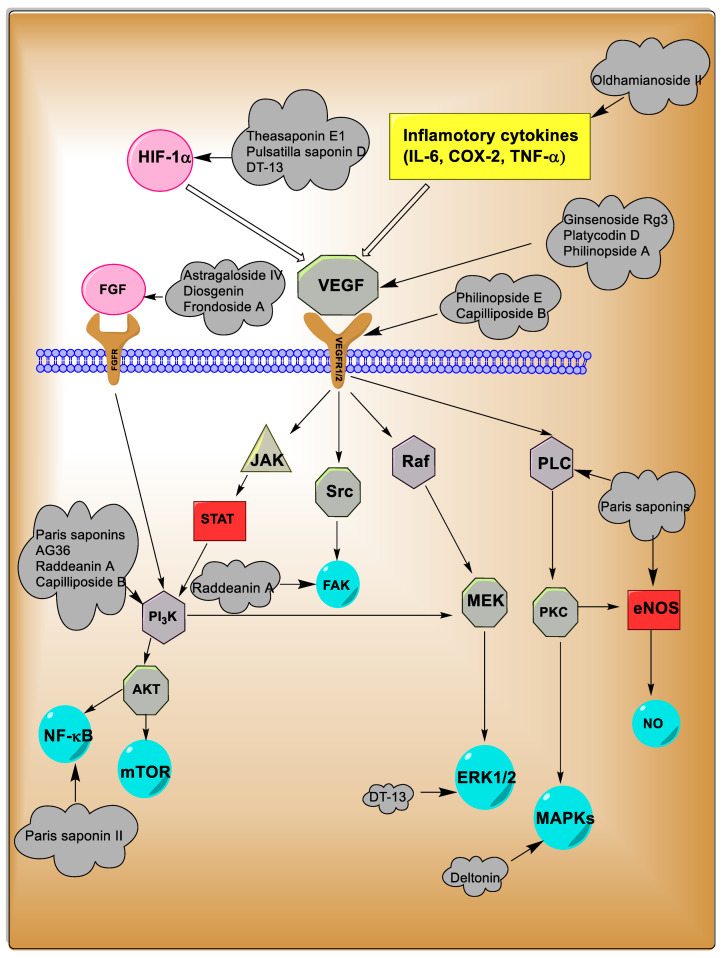
Anti-angiogenesis mechanisms of saponins.

Saponins are natural compounds with a unique and diverse chemical structure, which has led to numerous pharmacological and biological effects from these compounds. These compounds have noteworthy anti-cancer effects on different types of cancer. The inhibition of angiogenesis as one of the main approaches for cancer treatment has always been considered by researchers. Accordingly, saponins showed prominent anti-angiogenic effects by various mechanisms (Figure 6). The inhibition of VEGF and HIF-1α are among the most critical anti-angiogenesis mechanisms of saponins. These compounds exerted anti-angiogenic effects via inhibiting the signaling pathways, including MAPKs, FGF2, and PI3K/Akt/mTOR. Despite the fact that humans and animals widely consume plants containing saponins, these compounds have side effects, such as bleeding, digestive complications, hypertension, genotoxicity, kidney/liver necrosis, and hormonal interference in high doses [163,229,230,231,232]. Furthermore, due to the complexity of the chemical structure of saponins, there is a possibility of their interference with chemotherapy regimens, so more research is needed surrounding their use in cancer treatment [93]. Recently, extensive bioavailability studies (NCT04335435) on saponins have been designed in order to prescribe the appropriate dose and reduce their side effects and drug interactions. On the other hand, the isolation and purification of saponins from natural sources is complex and requires multiple techniques and time-consuming steps, so it is necessary to conduct more studies to find new ways to isolate and purify these compounds. Therefore, triterpenoids and steroidal saponins can be introduced as promising natural compounds for the treatment of cancer. For this purpose, extensive experimental and clinical trial studies are recommended.

## Figures and Tables

**Table 1:** Terpenoid and steroidal saponins with anti-angiogenesis activities.

Saponins	Anti-Angiogenesis Mechanisms	Administration Rout/Dosage	Natural Sources	References
Terpenoid saponin				
AG36	↓ VEGF and FAK/PI3K/Akt gene expression	In vivo study; 75 and 1.5 mg/kg/day/i.p. in vitro study; 20 5, 10, and 20 μM	*Ardisia gigantifolia* (Primulaceae)	[188]
Arenaroside C, D, E, and G	NR	In vitro study; <5 μM	*Polycarpaea arenaria* (Caryophyllaceae)	[189]
Ginsenoside-Rb1	↑ PEDF and PPAR-γ gene expression	In vitro study; 100–500 nM	*Panax* spp. (Araliaceae)	[192,193]
Ginsenoside-Rb2	NR	In vivo study; 100 µg/mouse, i.v.	*Panax* spp. (Araliaceae)	[194]
Ginsenoside K	↓ SPHK1 activities	In vitro study; 10 µg/mL	*Panax ginseng* (Araliaceae)	[196]
Ginsenoside Rg3	↓ VEGF gene expression	In vivo study; 20 mg/kg	*Panax* spp. (Araliaceae)	[197]
senegin III	↑ PEDF, inhibition VEGF activities	In vitro study; 0.1, 1, and 10 μM	*Polygala senega* (Polygalaceae)	[198]
Theasaponin E1	↓ VEGF, Akt, and HIF-1α gene expression	In vitro study; 2 μM and 10 μg/mL	*Camellia sinensis* (Theaceae)	[201,202]
Oldhamianoside II	↓ VEGF and VEGFR2, COX-2, bFGF gene expression, ↓ pro-inflammatory cytokines activities including TNF-α and IL-6	In vivo study; 5, 10, and 20 mg/kg, i.p.	*Gypsophila oldhamiana* (Caryophyllaceae)	[170,200]
Platycodin D	↓ VEGFR2, PLCγ1, JAK2, FAK, Src, and Akt gene expression	In vitro study; 0.3, 1, 3, 10, and 30 μM	*Platycodon grandiflorus* (Campanulaceae)	[209,210]
Julibroside J8	NR	In vivo study; 0.5, 1.5, and 3 mg/kg, p.o. In vitro study; 0.5, 1, 2, and 4 μg/mL. Ex vivo study 30 and 50 μg/egg	*Albizia julibrissin* (Fabaceae)	[211]
Maesasaponins (I-VII.1)		25 and 50 μg/mL for in vivo (zebrafish model) and in vitro studies. Ex vivo study 1 µg/pellet.	*Maesa lanceolata* (Myrsinaceae)	[212]
Astragaloside IV	↓ VEGF and FGF2 gene expression	In vivo study; 20 mg/kg, p.o.	*Astragalus membranaceus* (Fabaceae)	[213]
Capilliposide B	↓ VEGF, Erk, VEGFR2, and Akt gene expression	In vitro study; 0.25, 0.5, 1 μM	*Lysimachia capillipes* (Primulaceae)	[214]
Chiisanoside	↓ VEGF gene expression	In vivo study; 60, 120, and 240 mg/kg, i.p.	*Acanthopanax sessiliflorus* (Araliaceae)	[203]
Pulsatilla saponin D	↓ VEGF and HIF-1α gene expression	In vitro study; 10 μM	*Pulsatilla koreana* (Ranunculaceae)	[204,205,206,207]
Gleditsiosides B	↓ VEGF and bFGF gene expression	In vitro study; 1 μM	*Gleditsia sinensis* (Fabaceae)	[217]
Raddeanin A	↓ PLCγ 1, JAK2, FAK, Src, and Akt and VEGFR2 activities and ↓ Wnt/β-catenin gene expression	In vivo study; 100 mg/kg/day, i.p. in vitro study; 100, 200 nM, 0.1, 0.3, 1, and 3 μM. Ex vivo study; 0.3, 1, 3, and 10 μM.	*Anemone raddeana* (Ranunculaceae)	[215,216]
Philinopside E *	↓ (KDR)/VEGFR2 gene expression	In vivo study; 2 mg/kg and 3 mg/kg, i.v. In vitro study; 1.25, 2.5, and 5 μM. Ex vivo; 2.5, 5, 10, and 10 nM/egg	*pentacta quadrangularis* (Cucumariidae)	[219,220]
Philinopside A *	↓ VEGF, bFGF and PDGF gene expression	In vivo study; 1, 2, and 4 mg/kg, i.v. In vitro study; 50 μM. Ex vivo study, 10 nM/egg	*pentacta quadrangularis* (Cucumariidae)	[221]
Frondoside A *	↓ bFGF activities	In vivo study; 1 and 0.01 mg/kg/day, i.p. Ex vivo study 100 and 500 nM/egg	*Cucumaria frondose* (Cucumariidae)	[222]
Steroidal saponin				
Diosgenin	↓ VEGF and bFGF gene expression	In vitro study; 5, 15, and 25 µM.	*Dioscorea* spp. (Dioscoreaceae), *Trigonella foenum-graecum* (Fabaceae)	[177]
Deltonin	↓ VEGF, MAPK/AKT pathway activities	In vitro study; 1 and 4 μM	*Dioscorea zingiberensis* (Dioscoreaceae)	[178]
Terrestrosin D	Direct inhibition of vascular endothelial cell proliferation	In vivo study; 20 mg/kg, i.p.	*Tribulus terrestris* (Zygophyllaceae)	[180]
Paris saponin II	↓ NF-κB gene expression	In vivo study; 25 mg/kg, i.p. In vitro study 2.5 μM.	*Paris polyphylla* (Melanthiaceae)	[183,184]
Paris saponin I, II, Ⅵ and Ⅶ	↓ PI3K/AKT/mTOR/S6K1, SRC/eNOS, PLCγ/ERK and JAK2/STAT3 and R2 gene expression	In vitro study, 2 and 4 μM	*Paris polyphylla* (Melanthiaceae)	[181]
Polyphyllin D	Direct inhibition of vascular endothelial cell proliferation	In vivo study; 0.313 μM and 0.156 μM, zebrafish model. In vitro study, 200, 300, and 400 nM	*Paris polyphylla* *(Melanthiaceae)*	[182]
ACS	↓ MMP 2 and 9 gene expression	In vivo study; 5 μM, i.p. In vitro study 1.25, 2.5, and 5 μM	*Ophiopogon japonicas* (Asparagaceae)	[185]
DT-13	↓ ERK1/2, HIF-1α and Akt gene expression	In vitro study, 0.01, 0.1, and 1 μM. Ex vivo study; 100, 10, and 1 μmol/egg.	*Ophiopogon japonicas* (Asparagaceae)	[186]
Convallamaroside	NR	In vivo study; 5, 10, 20, 50, and 100 μg/mL, p.o. In vitro study; 10 and 50 μg/mL	*Convallaria majalis* (Asparagaceae)	[187]

bFGF: Basic fibroblast growth factor, COX-2: Cyclooxygenase-2, ERK: Extracellular signal-regulated kinase, FAK: Focal adhesion kinase, FGF2: Fibroblast growth factor 2, HIF-1α: Hypoxia-inducible factor 1-alpha, i.p.: Intraperitoneal, i.v.: intravenous, IL-6: Interleukin-6, JAK2: Janus kinase 2, MAPK: Mitogen-activated protein kinase, MMP: Matrix metalloproteinases, mTOR/S6K1: Mammalian target of rapamycin/ribosomal protein S6 kinase beta-1, NF-κB: Nuclear factor κ light chain enhancer of activated B cells, NR: Not report, p.o.: Oral administration (per os), PEDF: Pigment epithelium-derived factor, PI3K/Akt: Phosphoinositide 3-kinases/Protein kinase B, PLCγ: Phospholipase Cγ, PPAR-γ: Peroxisome proliferator-activated receptor gamma, SPHK1: Sphingosine kinase 1, SRC/eNOS: SRC kinase/endothelial nitric oxide synthase, STAT3: Signal transducer and activator of transcription 3, TNF-α: Tumor necrosis factor alpha, VEGF: Vascular endothelial growth factor, VEGFR2: VEGF receptor2, ↓: Reduce, ↑: Increase, *: Saponins isolated from marine organisms.
